# New Advances on the Dispersive and Polar Surface Properties of Poly(styrene-co-butadiene) Using Inverse Gas Chromatography

**DOI:** 10.3390/polym16233233

**Published:** 2024-11-21

**Authors:** Tayssir Hamieh

**Affiliations:** 1 Faculty of Science and Engineering, Maastricht University, P.O. Box 616, 6200 MD Maastricht, The Netherlands; t.hamieh@maastrichtuniversity.nl; 2 Institut de Science des Matériaux de Mulhouse, Université de Haute-Alsace, CNRS, IS2M UMR 7361, F-68100 Mulhouse, France; 3 Laboratory of Materials, Catalysis, Environment and Analytical Methods (MCEMA), Faculty of Sciences, Lebanese University, Beirut P.O. Box 6573/14, Lebanon

**Keywords:** retention volume, adsorption, organic solvents, Hamieh thermal model, London dispersive interaction energy, deformation polarizability, dispersive and polar surface energy, Lewis’s acid–base constants, molecular separation distance

## Abstract

The dispersive and polar properties of materials, and especially of polymers and copolymers, play an important role in several engineering applications implying their surfaces and interfaces. The surface energetic properties of poly(styrene-co-butadiene) have never been studied. We proposed in this study an accurate determination of such properties by using inverse gas chromatography (IGC) at infinite dilution. Background: The IGC surface technique led to the dispersive and polar properties of poly(styrene-co-butadiene) rubber (SBR) by adsorption of organic solvents at various temperatures. Methods: Our new methodology, based on the thermal Hamieh model and the London dispersion interaction energy, was used to determine the London dispersion surface energy, the polar acid–base surface energy, and the Lewis acid–base properties of the copolymer. Results: The different surface energy parameters of the SBR were obtained as a function of temperature from the chromatographic measurements. Conclusions: The dispersive and polar free energies of adsorption of the various n-alkanes and polar molecules on poly(styrene-co-butadiene) were determined at different temperatures. A decrease in the London dispersive surface energy and the polar Lewis acid–base surface energies of SBR was highlighted when the temperature increased. It showed a Lewis amphoteric character of poly(styrene-co-butadiene) with a highest basic constant 10 times larger than its acidic constant. This new and original method can better characterize the surface thermodynamic properties of poly(styrene-co-butadiene).

## 1. Introduction

One of the most widely used synthetic rubbers is poly(styrene-co-butadiene) rubber (SBR), considered the most interesting copolymer because of its low manufacturing cost and excellent properties, especially to replace natural rubber in practically all industrial uses and applications [1,2].

SBR materials are not only used in the manufacturing of tires but also in several engineering applications, such as roofing barriers, cover trips, wires, footwear, and sporting goods. They are also used in reinforced form with inorganic fillers [3,4,5,6,7,8,9]. The improvement of the properties of SBR rubber was developed in the literature. Radhakrishnan et al. [10] studied the thermal behavior of SBR/poly (ethylene-co-vinyl acetate) (EVA) blends. They improved SBR thermal stability by the addition of compatibilizer. Other works were devoted to the determination of the flammability of rubbers, the toxicity of the thermal decomposition, and the combustion products of the vulcanizates [11,12]. Vo et al. [13] measured the dielectric properties of styrene-butadiene rubber (SBR) composites and deduced the polymer relaxation at the polymer–nanoparticle interface. By adding some fillers, such as clays, silica, and carbon black, into SBR matrices, it was possible to optimize the mechanical properties [14,15,16,17,18,19,20,21,22,23,24,25].

Some chemical parameters of SBR were determined by several authors. Diez et al. [26] investigated the solubility, the Flory–Huggins interaction parameters of SBR, and the infinite dilution activity coefficients of various solvents by using the inverse gas chromatography technique. The heat of vaporization and the Hansen solubility parameter of binary solvent poly(styrene-block-butadiene) rubber systems were determined by Benguergoura et al. [27] by the investigation of both polar and nonpolar solvents using the IGC technique. Whereas Farshchi et al. [28] used the solubility parameter of SBR to predict the compatibility of solvents with the polymer and its interaction parameters.

However, the surface thermodynamic properties of the poly(styrene-co-butadiene), such as the free energy, the enthalpy and entropy of adsorption, the dispersive and polar surface energies, and the Lewis acid–base properties of this copolymer, were not accurately determined in the literature. This study was devoted to the application of our new methodology for the determination of the different surface thermodynamic parameters of poly(styrene-co-butadiene) using the inverse gas chromatography at infinite dilution [29,30,31,32,33,34,35,36,37,38,39]. This powerful technique experimentally allowed us to obtain the net retention time of the n-alkanes and polar solvents adsorbed on the copolymer at different temperatures. The obtained chromatographic measurements led to the expression of the London dispersive surface energy of SBR as a function of the temperature using the Hamieh thermal model [40,41,42,43] and Fowkes’s relation [44]. This new model proposed new expressions of the surface area of the adsorbed organic molecules versus the temperature [40] and allowed us to determine more accurate values of the London dispersive surface energy. Whereas the polar contributions of the different thermodynamic variables of adsorption were determined by applying the London interaction energy given as a function of the deformation polarizability of the various solvents and solid materials [45,46,47,48,49,50,51].

## 2. Materials and Methods

### 2.1. Solvents and Materials

The poly(styrene-co-butadiene) (${\bar{M}}_{n}=1.8\times 10^{5}\;g/mol$) and the different n-alkanes, and polar solvents were purchased from Sigma-Aldrich (Beirut, Lebanon). The n-alkanes were the following: n-pentane, n-hexane, n-heptane, n-octane, and n-nonane. Whereas the polar solvents characterized by their donor (*DN* in J/mol) and acceptor (*AN* in J/mol) numbers of electrons [52,53] were given as follows:

- The Lewis’s acid solvents such as: chloroform (*DN* = 0, *AN* = 22.54) and dichloromethane (*DN* = 5.02, *AN* = 16.27)
- The amphoteric solvents such as: benzene (*DN* = 0.42, *AN* = 0.72), ethanol (*DN* = 80.35, *AN* = 43.27), acetonitrile (*DN* = 59.01, *AN* = 19.65), and toluene (*DN* = 16.32, *AN* = 3.98)
- The Lewis’s base solvents such as: acetone (*DN* = 71.15, *AN* = 10.49), ethyl acetate (*DN* = 71.56, *AN* = 6.39), diethyl ether (*DN* = 80.35, *AN* = 5.91), cyclohexane (*DN* = 5.89, *AN* = 0.17), and tetrahydrofuran (THF) (*DN* = 83.70, *AN* = 2.29)

### 2.2. Inverse Gas Chromatography

A Focus GC gas chromatograph (Sigma-Aldrich, Lyon, France) equipped with a flame ionization detector of high sensitivity was used to carry out the measurements of the retention times of the organic solvents adsorbed on the SBR copolymer. A stainless-steel column, with a length of 30 cm and a 2 mm internal diameter, contained 0.5 g of SBR powder. The flow rate of helium used as a carrier gas was optimized to 20 mL/min. A Hamilton microsyringe with a volume of 1 μL was used for the injection of an infinite quantity of organic probes for temperatures varying from 30 °C to 70 °C. Each solvent injection was repeated three times to determine the average retention times, obtained with a standard deviation of 1%.

The net retention time of organic solvents adsorbed on the copolymer was obtained using the methane probe as an inert solvent and the first order integration. The net retention volume Vn(T) of the adsorbed solvents was directly deduced as a function of the temperature *T* from the net retention time measurements.

### 2.3. Thermodynamic Methods

The net retention volume of the organic probes adsorbed on SBR led to the free energy of adsorption $∆G_{a}^{0}(T)$ of the different solvents using the following equation:

(1) $$∆G_{a}^{0}\left(T\right)=-RTlnVn(T)+C(T)$$

where R is the perfect constant gas, and C(T) a thermodynamic parameter depending on the temperature and the interaction between the solvent and the polymer.

The free energy of adsorption can be written as follows:

(2) $$∆G_{a}^{0}\left(T\right)=∆G_{a}^{d}(T)+∆G_{a}^{p}(T)$$

where $∆G_{a}^{d}(T)$ and $∆G_{a}^{p}(T)$ are the London dispersive and polar interaction energies, respectively.

In the case of the adsorption of n-alkanes (non-polar solvents such as) on SBR, the free interaction energy is equivalent to London dispersive energy:

(3) $$∆G_{a}^{0}\left(T\right)=∆G_{a}^{d}(T)$$

The determination of the London dispersive component $\gamma_{s}^{d}$ of the surface energy of a solid material was first proposed by Dorris and Gray [54] using Fowkes’s relation [44] and correlating the work of adhesion $W_{a}$ to the free energy of adsorption by the help of the surface area *a* of the adsorbed molecule and the geometric mean of the dispersive components of the liquid solvent $\gamma_{l}^{d}$ and the solid $\gamma_{s}^{d}$

(4) $$∆G_{a}^{0}=Na\;W_{a}=2Na\;\sqrt{\gamma_{l}^{d}\gamma_{s}^{d}}$$

where N is Avogadro’s number.

Dorris and Gray obtained the dispersive component of the surface energy of a solid by taking the following increment $∆G_{-CH2-}^{0}$ given by Equation (5):

(5) $$∆G_{-CH2-}^{0}=∆G^{0}\left(C_{n+1}H_{2(n+2)}\right)-∆G^{0}\left(C_{n}H_{2(n+1)}\right)$$

where $C_{n}H_{2(n+1)}$ and $C_{n}H_{2(n+1)}$ represent the general formula of two consecutive n-alkanes.

The London dispersive surface energy $\gamma_{s}^{d}$ can be then determined by Equation (6):

(6) $$\gamma_{s}^{d}=\frac{{\left[RTln\left[\frac{V_{n}\left(C_{n+1}H_{2(n+2)}\right)}{V_{n}\left(C_{n}H_{2(n+1)}\right)}\right]\right]}^{2}}{4N^{2}a_{-CH2-}^{2}\gamma_{-CH2-}}$$

Dorris and Gray used the surface area of methylene group with $a_{-CH2-}=6Å$, independent from the temperature, and the surface energy $\gamma_{-CH2-}$ of methylene group given by the following Equation:

(7) $$\gamma_{-CH2-}\left(in\;mJ/m^{2}\right)=52.603-0.058\;T\left(T\;in\;K\right)$$

A similar method to determine $\gamma_{s}^{d}$ was also proposed in the literature [31] (Equation (8)):

(8) $$RTln(Vn)=2Na{\left(\gamma_{l}^{d}\gamma_{s}^{d}\right)}^{1/2}+\alpha (T)$$

where α(T) is a constant only depending on the temperature and the solid surface.

In the above methods, the London dispersive surface energy of the solvents $\gamma_{l}^{d}$ and the surface areas of the molecules and methylene group were supposed to be independent from the temperature. Other studies [37] proposed a correction of $\gamma_{l}^{d}(T)$ and the use of different molecular models of the surface areas of organic molecules, such as the geometric model, cylindrical model, spherical model, Kiselev results, Van der Waals (VDW) equation, and the Redlich-Kwong (R-K) equation [37].

In 2020, we proposed new relations of the surface area $a\left(T\right)$ of polar and non-polar molecules and the expression of surface area of methylene group $a_{-CH2-}\left(T\right)$ as a function of the temperature to correct the calculation of the London dispersive surface energy of solid materials [40].

The determination of the polar free energy and the polar enthalpy of adsorption was performed by several authors using different reference thermodynamic parameters, such as the boiling point of the solvents [29], the vapor pressure of the probes at a fixed temperature [32,33], the London dispersive surface energy $\gamma_{l}^{d}$ [31], the deformation polarizability [34], or the topological index $\chi_{T}$ [35,36]. However, we showed that these various methods cannot be considered as accurate quantitative methods. The only method theoretically well-founded was that based on the deformation polarizability. However, this method was not correctly used in the literature because of several approximations leading to wrong values of the polar free energy. Recently, we proposed the London dispersion interaction energy given by Equation (9):

(9) $$∆G_{a}^{d}\left(T\right)=-\frac{\alpha_{0S}\;}{\;H^{6}}\left[\frac{3N}{{2\left(4\pi \varepsilon_{0}\right)}^{2}}\left(\frac{\varepsilon_{S}\;\varepsilon_{X}}{\left(\varepsilon_{S}+\varepsilon_{X}\right)}\alpha_{0X}\right)\right]$$

where $\varepsilon_{0}$ is the dielectric constant of vacuum, $\alpha_{0S}\;$ and $\alpha_{0X}$ the respective deformation polarizabilities of the solid material denoted by S and the organic solvent denoted by X, separated by a distance H, and $\varepsilon_{S}$ and $\varepsilon_{X}$ their corresponding ionization energies.

Using the previous equations, Equation (9) can be written as follows:

(10) $$RTlnVn=\frac{\alpha_{0S}\;}{\;H^{6}}\left[\frac{3N}{{2\left(4\pi \varepsilon_{0}\right)}^{2}}\left(\frac{\varepsilon_{S}\;\varepsilon_{X}}{\left(\varepsilon_{S}+\varepsilon_{X}\right)}\alpha_{0X}\right)\right]-∆G_{a}^{p}\left(T\right)+C\left(T\right)$$

A new chromatographic interaction parameter $P_{SX}$ was therefore proposed:

(11) $$P_{SX}=\frac{\varepsilon_{S}\;\varepsilon_{X}}{\left(\varepsilon_{S}+\varepsilon_{X}\right)}\alpha_{0X}$$

The variations of RTlnVn of n-alkanes can be then given by Equation (12):

(12) $$\left\{\begin{array}{c} RTlnVn\left(non-polar\right)=A\left[\frac{3N}{{2\left(4\pi \varepsilon_{0}\right)}^{2}}P_{SX}\left(non-polar\right)\right]+C(T)\\ A=\frac{\alpha_{0S}\;}{\;H^{6}}\;\;\;\;\;\;\;\;\;\;\;\;\;\;\;\;\;\;\;\;\;\;\;\;\; \end{array}\right.$$

where A is a dispersive parameter of the polymer.

The free polar energy $∆G_{a}^{p}\left(polar\right)$ of the adsorbed polar solvents on the SBR can be obtained by the following equation:

(13) $$∆G_{a}^{p}\left(T,\;polar\right)=RTlnVn\;\left(T,\;polar\right)-A\left[\frac{3N}{{2\left(4\pi \varepsilon_{0}\right)}^{2}}P_{SX}\left(polar\right)\right]-C(T)$$

The values of $∆G_{a}^{p}\left(T,\;polar\right)$ obtained by Equation (13) versus the temperature led to the enthalpy $\left(-∆H_{a}^{p}\right)$ and entropy $\left(-∆S_{a}^{p}\right)$ of adsorption of polar solvents on the copolymer using Equation (14):

(14) $$∆G_{a}^{p}\left(T\right)=∆H_{a}^{p}-T∆S_{a}^{p}$$

The determination of the enthalpic (*K_A_*, *K_D_)* and entropic ($\omega_{A}$, $\omega_{D}$) Lewis’s acid–base constants of the PBR copolymer were determined by Equation (15):

(15) $$\left\{\begin{array}{c} \left(-∆H^{p}\right)=\;K_{A}\times DN+K_{D}\times AN\;\;\\ \left(-∆S_{a}^{p}\right)=\omega_{A}\times DN+\omega_{D}\times AN \end{array}\right.$$

where DN and AN are, respectively, the corrected electron donor and acceptor numbers of the polar molecule.

The London dispersive surface energy $\gamma_{s}^{d}(T)$ of the PBR as a function of the temperature was obtained using the Hamieh thermal model. Whereas the polar (or acid–base) contribution of the surface energy $\gamma_{s}^{p}=\gamma_{s}^{AB}$ of the poly(styrene-co-butadiene) was calculated by applying the Van Oss et al.’s method [55] (Equation (16)):

(16) $$\gamma_{s}^{p}=2\sqrt{\gamma_{s}^{+}\gamma_{s}^{-}}$$

where $\gamma_{s}^{+}$ and $\gamma_{s}^{-}$ are the Lewis acid and base surface energies of the solid material, respectively.

The Van Oss et al. [55] method used two solvents such as ethyl acetate (base *B*) and dichloromethane (acid *A*), characterized by the following parameters:

(17) $$\left\{\begin{array}{c} \gamma_{A}^{+}=5.2\;mJ/m^{2},\;\gamma_{A}^{-}=0\;\;\;\;\;\\ \gamma_{B}^{+}=0,\;{\;\gamma }_{B}^{-}=19.2\;mJ/m^{2} \end{array}\right.$$

Using the expression $∆G_{a}^{p}\left(T\right)$ of the polar solvents given by:

(18) $$∆G_{a}^{p}\left(T\right)=2Na(T)\left(\sqrt{\gamma_{l}^{-}\gamma_{s}^{+}}+\sqrt{\gamma_{l}^{+}\gamma_{s}^{-}}\right)$$

We deduced the Lewis acid and base surface energies of the copolymer by Equation (19):

(19) $$\left\{\begin{array}{c} \begin{array}{c} \gamma_{s}^{+}\left(T\right)\;=\frac{{\left[∆G_{a}^{p}\left(T\right)\left(B\right)\right]}^{2}}{{4N}^{2}{\left[a_{B}(T)\right]}^{2}\gamma_{B}^{-}}\; \end{array}\;\\ \gamma_{s}^{-}\left(T\right)=\frac{{\left[∆G_{a}^{p}\left(T\right)\left(A\right)\right]}^{2}}{{4N}^{2}{\left[a_{A}(T)\right]}^{2}\gamma_{A}^{+}}\; \end{array}\right.$$

Therefore, the total surface energy $\gamma_{s}^{tot.}$ of the SBR can be obtained from Equation (20):

(20) $$\gamma_{s}^{tot.}=\gamma_{s}^{d}+\gamma_{s}^{p}$$

## 3. Experimental Results

### 3.1. London Dispersive Surface Energy of the SBR Copolymer

**Figure 1:**
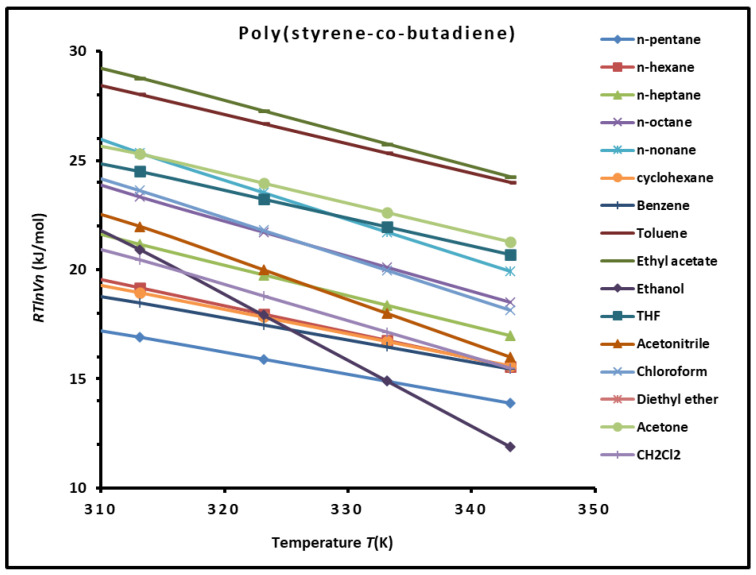
Variations of RTlnVn (kJ/mol) of organic solvents adsorbed on poly(styrene-co-butadiene) against the temperature.

The chromatographic measurements led to the values of RTlnVn(T) for the adsorbed solvents as a function of the temperature. The results are given in Table S1 (Supplementary Materials). In Figure 1, we show the evolution of RTlnVn(T) for the different organic solvents adsorbed on the SBR versus the temperature.

The variations of RTlnVn for the adsorbed organic solvents adsorbed on the PBR, as shown in Figure 1, showed linear evolution for the solvents. Indeed, the temperature interval is located outside of the transition temperatures of the copolymer. This justified the linear variations of the thermodynamic parameter, such as RTlnVn, as a function of temperature.

**Figure 2:**
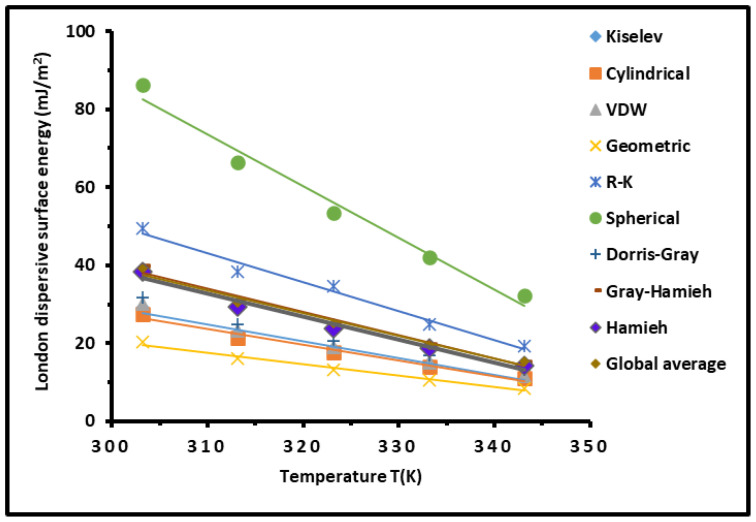
Variations of the London dispersive surface energy $\gamma_{s}^{d}\;(mJ/m^{2})$ of SBR as a function of the temperature *T* (K) using the different molecular models compared to Hamieh thermal model.

The representation of RTlnVn given in Table S1 versus $2Na(T)\;\sqrt{\gamma_{l}^{d}(T)}$ of n-alkanes adsorbed on SBR at different temperatures led to linear variation of the London dispersive surface energy $\gamma_{s}^{d}(T)$ using the variations of the surface area a(T) of solvents obtained from the Hamieh thermal model [40]. The results of $\gamma_{s}^{d}(T)$ from the SBR, obtained by applying the other molecular models and that used by Schultz et al. [31], were compared in Figure 2 to those of the Hamieh thermal model [40].

Figure 2 showed large differences in the values of the London dispersive surface energy of the SBR obtained from the use of the various molecular models. The highest values of $\gamma_{s}^{d}$ were obtained by the spherical model, representing an overestimation of the dispersive surface energy, while the smallest values were those of the geometric model. Whereas the accurate values were obtained by the Hamieh thermal model [40] that took into account the thermal effect on the surface area of the organic molecules. The global average was obtained by taking the mean average of all the models coinciding with the values of the thermal model.

The deviation between the various molecular models and the Hamieh thermal model can be clarified by determining the equations of $\gamma_{s}^{d}(T)$ of the copolymer versus the temperature. The results given in Table 1 include the values of the dispersive surface entropy $\varepsilon_{s}^{d}$, the extrapolated values $\gamma_{s}^{d}(T=0K)$ and the maximum of temperature $T_{Max}$ defined by: $T_{Max}=-\frac{\gamma_{s}^{d}(T=0K)}{\varepsilon_{s}^{d}}$ for SBR.

The results given in Table 1 showed that the Hamieh thermal model applied to the Dorris–Gray method and Fowkes’s equation gave identical values of $\varepsilon_{s}^{d}$, $\gamma_{s}^{d}(T=0K)$, and $T_{Max}$ compared to the averages values, whereas a large deviation was observed with the other models. However, all the molecular models gave approximately the same values of the maximum temperature $T_{Max}=367.5\pm 2.5\;K$. This temperature can be considered as an important intrinsic thermodynamic characteristic of the copolymer.

### 3.2. Variations of Polar Free Surface Energy ($-∆G_{a}^{p}\left(T\right)$) of SBR Copolymer

Using the Equations (9)–(14) relative to the adsorption of n-alkanes and polar molecules on SBR copolymer, we obtained the variations of the polar free surface energy ($-∆G_{a}^{p}\left(T\right)$) of the polar solvents versus the temperature. The results given in Table S2 allowed us to classify the polar solvents in increasing order by their polar free interaction energy ($-∆G_{a}^{p}\left(T\right)$) as follows:

Cyclohexane < Benzene < Dichloromethane < Chloroform < Toluene < THF < Ethanol < Acetonitrile < Ethyl acetate < Acetone < Diethyl ether

**Figure 3:**
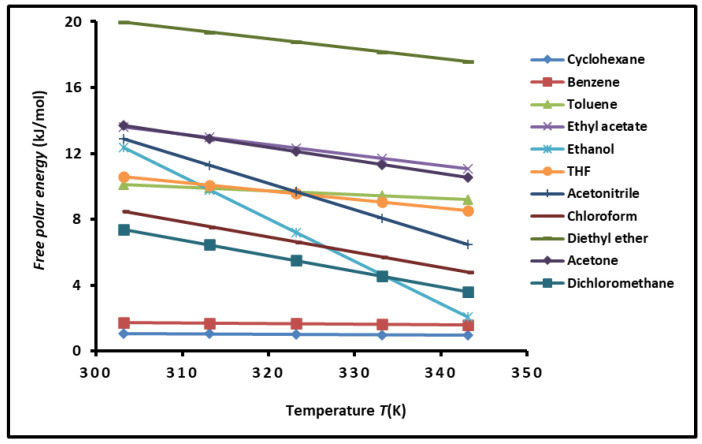
Variations of the polar free interaction energy $\left(-∆G_{a}^{p}\left(T\right)\right)$ (kJ/mol) of polar organic molecules adsorbed on SBR copolymer as a function of temperature.

The above classification varied slightly when the temperature increases from 303.15 K to 343.15 K for solvents such as THF, ethanol, and acetonitrile. The results showed that the highest value of ($-∆G_{a}^{p}\left(T\right)$) was obtained with diethyl ether, proving the highest basic character of the copolymer (Figure 3).

The curves plotted in Figure 3 showed linear variations of ($-∆G_{a}^{p}\left(T\right)$). This linearity is due to the temperature interval that was located outside of the transition temperature of SBR.

The different equations of ($-∆G_{a}^{p}\left(T\right)$) for the polar molecules are given in Table 2. This allowed us to obtain the polar enthalpy and entropy of adsorption of the various polar solvents using Equation (14) and consequently led to the Lewis acid–base constants of the copolymer.

### 3.3. Lewis Acid–Base Constants of Poly(styrene-co-butadiene)

The equations of ($-∆G_{a}^{p}\left(T\right))$ given in Table 2 led to the values of the polar enthalpy and entropy of adsorption of the adsorbed polar molecules. The results are given in Table 3.

**Figure 4:**
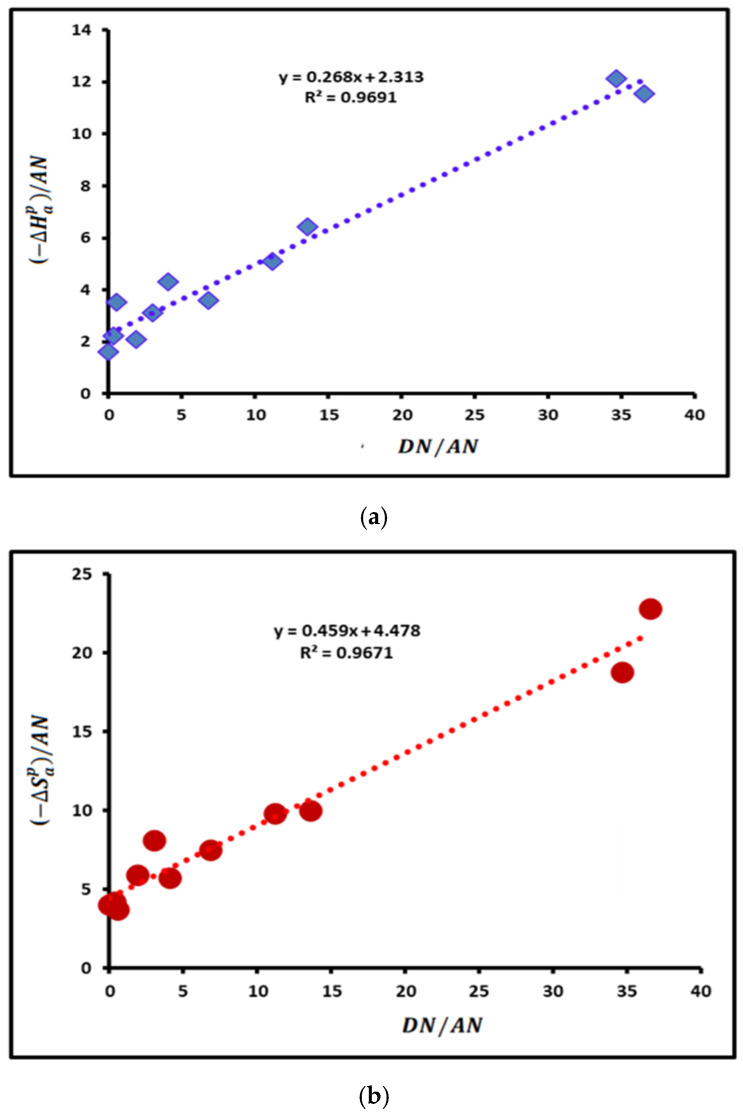
Variations of ($-∆H_{a}^{p})/AN$ (**a**) and ($-∆S_{a}^{p}\left(T)\right)/AN$ (**b**) of the various organic solvents adsorbed on SBR as a function of the ratio of donor number on acceptor number of electrons DN/AN of the polar molecules.

The values in Table 3 allowed us to draw, in Figure 4, the variation of ($-∆H_{a}^{p})/AN$ and ($-∆S_{a}^{p}\left(T)\right)/AN$ of the adsorbed organic molecules as a function of their DN/AN.

The linear variations of ($-∆H_{a}^{p})/AN$ and ($-∆S_{a}^{p}\left(T)\right)/AN$ shown in Figure 4 led to the values of the enthalpic and entropic Lewis acid–base constants of the SBR copolymer using Equation (15). The results given in Table 4 clearly showed the highest basic character of the poly(styrene-co-butadiene). The highest donor capacity of electrons of SBR led to a Lewis basicity which is about 8.6 times larger than its acidity. A similar result was observed in the values of the entropic Lewis acid and base of SBR.

The comparison of the results obtained with the poly(styrene-co-butadiene) to those obtained in a recent study [56] with another copolymer, such as polystyrene-*b*-poly(4-vinylpyridine) (PS-*b*-P4VP), showed that SBR is 3.5 times more basic and 2.8 more acidic than PS-*b*-P4VP. This is certainly due to the presence of butadiene in the SBR copolymer exhibiting larger Lewis acid–base constants.

### 3.4. Dispersive Free Energy and Interaction Distance Between the Solvents and SBR Copolymer

**Figure 5:**
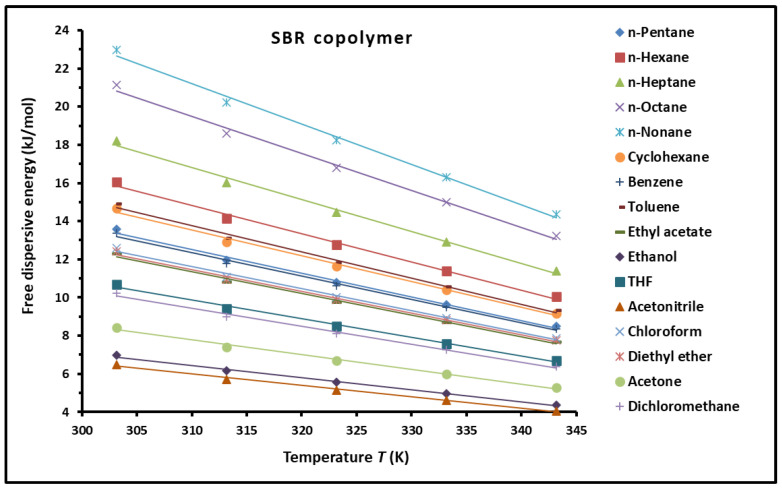
Variations of the free dispersive energy ($-∆G_{a}^{d}\left(T\right)$) of the different organic solvents adsorbed on the SBR as a function of the temperature *T* (K).

Using the London interaction equation and the chromatographic data, we determined the dispersive free energy of interaction between the different solvents and the copolymer. The results given in Table S3 were plotted in Figure 5.

The curves in Figure 5 showed the linear variations of the free dispersive energy ($-∆G_{a}^{d}\left(T\right))$ of all the adsorbed organic solvents versus the temperature *T* (K). The highest value of the free dispersive energy was obtained in the case of n-alkanes exhibiting the highest carbon atom number.

**Figure 6:**
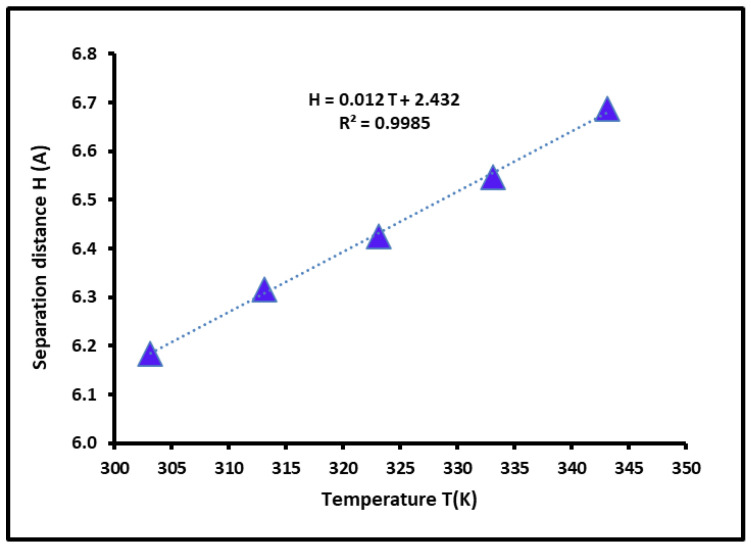
Variations of the interaction distance *H* (*T*) (Å) of the SBR as a function of temperature *T* (K).

The values of the free dispersive energy of adsorption led to the interaction distance H between the solvents and the copolymer. In Figure 6, we plotted the evolution of H(T) against the temperature. A linear variation of H(T) was obtained. The separation distance increased when the temperature increased. This is totally in good agreement with the concept of thermal agitation.

### 3.5. Determination of Polar Acid–Base Surface Energies and Total Surface Energy of the Copolymer

**Figure 7:**
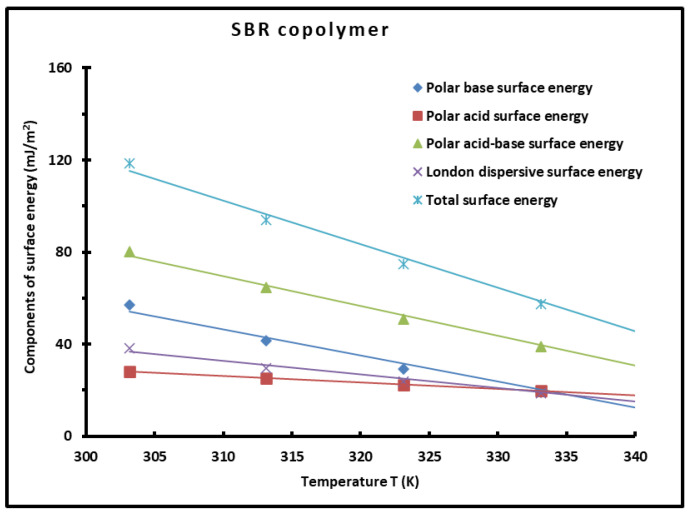
Variations of the polar acid and base surface energies $\gamma_{s}^{+}$, $\gamma_{s}^{-}$, the polar surface energy $\gamma_{s}^{p}$, the London dispersive surface energy $\gamma_{s}^{d}$, and the total surface energy $\gamma_{s}^{tot.}$ (mJ/m^2^) of the SBR as a function of the temperature.

The values of the polar Lewis acid $\gamma_{s}^{+}$ and base $\gamma_{s}^{-}$ surface energies of the SBR copolymer with respect to the temperature were obtained by applying the Van Oss et al.’s method [55] (Equations (16)–(19)). The polar acid–base surface energy $\gamma_{s}^{AB}=\gamma_{s}^{p}$ of the copolymer was determined using Equation (20). Whereas the total surface energy of the copolymer $\gamma_{s}^{tot.}$ was obtained by summing the two polar and dispersive contributions. The values of the different polar acid and base surface energies of the SBR given in Table 5 allowed us to draw their variations in Figure 7 as a function of temperature.

The comparison between the different surface energy components of the copolymer showed the highest values of polar acid surface energy were due to the highest basic character of the surface groups of the copolymer blocks. The results in Table 4 showed that the London dispersive surface energy is equivalent to the half of the polar surface energy of the PS-*b*-P4VP diblock copolymer, whereas the lowest surface energy was obtained for the basic surface energy component, because of the weaker acid force of the surface groups of the copolymer.

The variations of the various surface energy components are plotted in Figure 7.

Figure 7 clearly shows linear variations of the surface energy components of the SBR copolymer. This linearity is a direct consequence of non-existence of transition temperatures in the interval temperature [300 K, 350 K]. In several previous works, a non-linear variation of the different components of the surface energy of polymers observed around the transition temperatures was proved by the presence of a maximum surface energy at these temperatures [49,50,51,56].

Furthermore, knowing the polar free energy $\left(-∆G_{a}^{p}\left(X\right)\right)$ of a polar molecule (X) with a surface area $a_{X}$, and the polar surface energy $\gamma_{s}^{p}$ of the SBR copolymer, we determined the polar surface energy $\gamma_{l}^{p}(X)$ of the organic molecules using Equation (21):

(21) $$\gamma_{l}^{p}(X)=\frac{{\left(-∆G_{a}^{p}\left(X\right)\right)}^{2}}{4N^{2}{a_{X}}^{2}\gamma_{s}^{p}}$$

**Figure 8:**
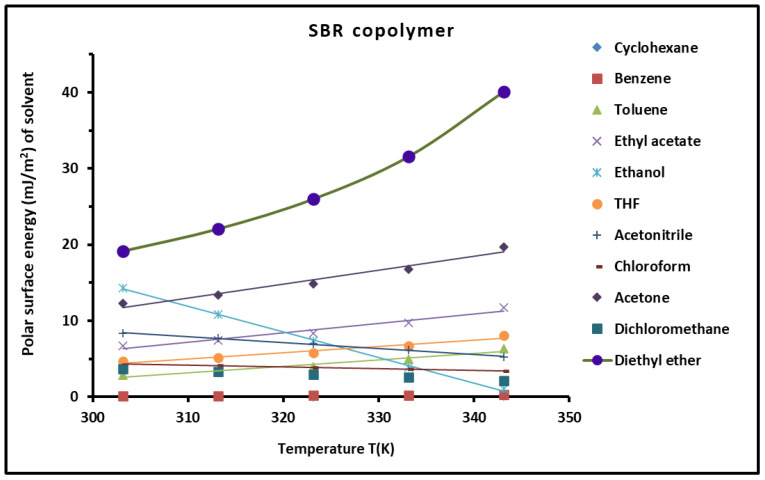
Evolution of the polar component of the surface energy $\gamma_{l}^{p}(T)$ (mJ/m^2^) of polar solvents adsorbed on the SBR copolymer as a function of temperature.

The polar surface energy $\gamma_{l}^{p}(X)$ of the solvents adsorbed on SBR was then calculated for the different temperatures and represented in Figure 8. An increase in the polar surface energy of the solvents was observed when the temperature increased, except for ethanol where a decrease of $\gamma_{l}^{p}$ was found. The results in Figure 8 showed that the highest value of $\gamma_{l}^{p}$ was obtained with diethyl ether and acetone adsorbed on SBR, due to the highest basic character of the copolymer.

## 4. Conclusions

The inverse gas chromatography technique at infinite dilution was utilized for the determination of the surface energetic properties of poly(styrene-co-butadiene) as a function of temperature. Several molecular models were applied to calculate the London dispersive surface energy of the SBR copolymer. The obtained results were corrected by applying the Hamieh thermal model, taking into account the temperature effect. A linear relation of the London dispersive surface energy $\gamma_{s}^{d}(T)$ of the SBR was obtained as a function of temperature, showing a decrease in the values of $\gamma_{s}^{d}(T)$ when the temperature increased. An intrinsic maximum temperature of the copolymer equal to 365.5 K was observed.

The London interaction equation was used to quantify the polar free energy, the polar enthalpy, and the polar entropy of the organic solvents adsorbed on the poly(styrene-co-butadiene). This recently proved to be the more accurate chromatographic method to determine the physicochemical properties of solid materials such as the Lewis acid–base parameters and the polar acid–base surface energy of polymers. The results showed that the SBR copolymer is 8.63 times more basic than acidic, with an acidic constant equal to 2.313 and a basic constant of 0.268. They also showed an interaction distance, *H*, between the solvents and the copolymer increasing with the temperature and varying from 6 Å to 6.9 Å, governed by the following equation $H\left(T\right)=0.012T+2.432$.

The application of our new method led to values of the acid $\gamma_{s}^{+}$ and base $\gamma_{s}^{-}$ surface energies, the polar acid–base surface energy $\gamma_{s}^{p}$, the London dispersive surface energy $\gamma_{s}^{d}$, and the total surface energy $\gamma_{s}^{tot.}$ of the copolymer as a function of temperature showing a decrease when the temperature increased.

## Figures and Tables

**Table 1:** Equations $\gamma_{s}^{d}(T)$ of SBR obtained by the various molecular models compared to the Hamieh thermal model, $\varepsilon_{s}^{d}$, $\gamma_{s}^{d}(T=0K)$, and $T_{Max}$.

Molecular Model	$\gamma_{s}^{d}(T)$ (mJ/m^2^)	$\varepsilon_{s}^{d}={d\gamma }_{s}^{d}/dT$ (mJ m^−2^ K^−1^)	$\gamma_{s}^{d}(T=0K)$ (mJ/m^2^)	$T_{Max}$ (K)	R^2^
Kiselev	$\gamma_{s}^{d}(T)$ = −0.43 T + 158.83	−0.43	158.83	367.66	0.9809
Cylindrical	$\gamma_{s}^{d}(T)$ = −0.40 T + 148.22	−0.40	148.22	368.61	0.9815
VDW	$\gamma_{s}^{d}(T)$ = −0.45 T + 165.22	−0.45	165.22	367.40	0.9808
Geometric	$\gamma_{s}^{d}(T)$ = −0.29 T + 108.25	−0.29	108.25	370.09	0.9821
Redlich-Kwong	$\gamma_{s}^{d}(T)$ = −0.74 T + 271.53	−0.74	271.53	368.28	0.9811
Spherical	$\gamma_{s}^{d}(T)$ = −1.32 T + 483.45	−1.32	483.45	365.59	0.9795
Dorris–Gray	$\gamma_{s}^{d}(T)$ = −0.45 T + 165.93	−0.45	165.93	371.13	0.9817
Gray-Hamieh	$\gamma_{s}^{d}(T)$ = −0.60 T + 219.89	−0.60	219.89	366.91	0.9799
Hamieh	$\gamma_{s}^{d}(T)$ = −0.59 T + 215.36	−0.59	215.36	365.45	0.9793
Global average	$\gamma_{s}^{d}(T)$ = −0.59 T + 215.19	−0.59	215.19	367.5	0.9824

**Table 2:** Equations of the polar free energy ($-∆G_{a}^{p}\left(T\right))$ as a function of temperature for the different polar solvents adsorbed on SBR.

Probes	Equation of ($-∆G_{a}^{p}\left(T\right)$ (kJ/mol))
Cyclohexane	($-∆G_{a}^{p}\left(T\right)$) = −0.0032T + 2.060
Benzene	($-∆G_{a}^{p}\left(T\right)$) = −0.0027T + 2.542
Toluene	($-∆G_{a}^{p}\left(T\right)$) = −0.023T + 17.108
Ethyl acetate	$(-∆G_{a}^{p}\left(T\right)$) = −0.0627T + 32.598
Ethanol	($-∆G_{a}^{p}\left(T\right)$) = 0.2574T +90.377
THF	($-∆G_{a}^{p}\left(T\right)$) = −0.0522T + 26.434
Acetonitrile	($-∆G_{a}^{p}\left(T\right)$) = −0.1103T + 91.466
Chloroform	($-∆G_{a}^{p}\left(T\right)$) = 0.0918T + 76.300
Diethyl ether	($-∆G_{a}^{p}\left(T\right)$) = −0.0593T + 37.928
Acetone	($-∆G_{a}^{p}\left(T\right)$) = −0.0791T + 57.669
Dichloromethane	($-∆G_{a}^{p}\left(T\right)$) = −0.0689T + 36.204

**Table 3:** Equations of the polar enthalpy ($-∆H_{a}^{p}$) and entropy ($-∆S_{a}^{p}$) of adsorption polar solvents on SBR copolymer as a function of temperature.

Probes	$\left(-∆S_{a}^{p}\right)$ (J/k.mol)	$\left(-∆H_{a}^{p}\right)$ (kJ/mol)
Cyclohexane	3.2	2.060
Benzene	2.7	2.542
Toluene	23.0	17.108
Ethyl acetate	62.7	32.598
Ethanol	257.4	90.377
THF	52.2	26.434
Acetonitrile	160.3	61.466
Chloroform	91.8	36.300
Diethyl ether	59.3	37.928
Acetone	79.1	37.669
Dichloromethane	68.9	36.204

**Table 4:** Values of the various enthalpic $(K_{A}$,$K_{D}$) and entropic $(\omega_{A}$,$\omega_{A})$ Lewis’s acid–base constants of the SBR copolymer, with the corresponding linear regression coefficients.

Lewis’s Acid–Base Parameter	Values	R^2^
$K_{A}$	0.268	0.9691
$K_{D}$	2.313	0.9691
$K_{D}$ */* $K_{A}$	8.631	0.9691
$\left(K_{D}+K_{A}\right)$	2.581	0.9691
$10^{3}\times \omega_{A}\;$	0.459	0.9671
$10^{3}\times \omega_{D}$	4.478	0.9671
$\omega_{D}/\omega_{A}$	9.756	0.9671
$10^{3}\times (\omega_{D}+\omega_{A})$	4.937	0.9671

**Table 5:** Values of the polar acid and base surface energies $\gamma_{s}^{+}$, $\gamma_{s}^{-}$, the polar surface energy $\gamma_{s}^{p}$, the London dispersive surface energy $\gamma_{s}^{d}$, and the total surface energy $\gamma_{s}^{tot.}$ (mJ/m^2^) of the SBR copolymer versus the temperature with the corresponding equations.

T(K)	303.15	313.15	323.15	333.15	343.15	Equation γ(T)
$\gamma_{s}^{-}$	56.95	41.59	29.09	19.17	11.57	$\gamma_{s}^{-}(T)$ = −1.132T + 397.42
$\gamma_{s}^{+}$	28.17	25.1	22.26	19.65	17.25	$\gamma_{s}^{+}(T)$ = −0.273T + 110.70
$\gamma_{s}^{p}$	80.11	64.62	50.9	38.82	28.25	$\gamma_{s}^{p}(T)$ = −1.295T + 471.08
$\gamma_{s}^{d}$	38.36	29.47	23.75	18.69	14.28	$\gamma_{s}^{d}(T)$ = −0.590T + 215.36
$\gamma_{s}^{tot.}$	118.47	94.08	74.65	57.5	42.53	$\gamma_{s}^{tot.}(T)$ = −1.885T + 686.43

## Data Availability

The original contributions presented in the study are included in the article/Supplementary Materials, further inquiries can be directed to the corresponding author.

## Supplementary Materials

The following supporting information can be downloaded at: https://www.mdpi.com/article/10.3390/polym16233233/s1, Table S1. Values of RTlnVn (kJ/mol) of solvents adsorbed on SBR as a function of the temperature. Table S2. Variations of the polar free energy $∆G_{a}^{p}\left(T\right)\;(kJ/mol)$ of different polar solvents adsorbed on SBR copolymer as a function of temperature. Table S3. Variations of dispersive free energy $∆G_{a}^{d}\left(T\right)\;(kJ/mol)$ of different solvents adsorbed on SBR copolymer as a function of temperature.
