# Supplementary material for: New Advances on the Dispersive and Polar Surface Properties of Poly(styrene-co-butadiene) Using Inverse Gas Chromatography
Source: Polymers (Basel). 2024 Nov 21;16(23):3233. doi: 10.3390/polym16233233 (PMC11644712; doi:10.3390/polym16233233)
Supplement: Supplementary file 1 [file polymers-16-03233-s001.zip › polymers-3306744-supplementary.pdf]

## Supplementary Materials

# New Advances on the Dispersive and Polar Surface Properties of Poly(styrene-co-butadiene) using Inverse Gas Chromatography

**Tayssir Hamieh[1,2,3]***

[1]Faculty of Science and Engineering, Maastricht University, P.O. Box 616, 6200 MD Maastricht, Netherlands.
[2]Université de Haute-Alsace, Institut de Science des Matériaux de Mulhouse, CNRS, IS2M UMR 7361, F-68100 Mulhouse, France
[3]Laboratory of Materials, Catalysis, Environment and Analytical Methods (MCEMA), Faculty of Sciences, Lebanese University, Hadath, Lebanon.

*Correspondence: Faculty of Science and Engineering, Maastricht University, P.O. Box 616, 6200 MD Maastricht, The Netherlands, E-mail: t.hamieh@maastrichtuniversity.nl

**Table S1.** Values of $RTlnVn$ (kJ/mol) of solvents adsorbed on SBR as a function of the temperature.

| Solvents | 303.15K | 313.15K | 323.15K | 333.15K | 343.15K |
|---|---|---|---|---|---|
| n-pentane | 17.800 | 16.898 | 15.898 | 14.898 | 13.898 |
| n-hexane | 20.352 | 19.164 | 17.964 | 16.764 | 15.564 |
| n-heptane | 22.605 | 21.151 | 19.761 | 18.372 | 16.984 |
| n-octane | 25.097 | 23.336 | 21.724 | 20.115 | 18.511 |
| n-nonane | 27.402 | 25.352 | 23.531 | 21.717 | 19.910 |
| cyclohexane | 20.015 | 18.958 | 17.841 | 16.723 | 15.605 |
| Benzene | 19.370 | 18.472 | 17.469 | 16.465 | 15.460 |
| Toluene | 29.321 | 28.035 | 26.697 | 25.360 | 24.022 |
| Ethyl acetate | 30.148 | 28.780 | 27.271 | 25.759 | 24.246 |
| Ethanol | 23.597 | 20.917 | 17.916 | 14.909 | 11.895 |
| THF | 25.576 | 24.504 | 23.236 | 21.965 | 20.690 |
| Acetonitrile | 23.628 | 21.978 | 19.990 | 17.996 | 15.995 |
| Chloroform | 25.322 | 23.628 | 21.802 | 19.975 | 18.146 |
| Diethyl ether | 36.626 | 35.278 | 33.792 | 32.305 | 30.816 |
| Acetone | 26.381 | 25.312 | 23.970 | 22.623 | 21.271 |
| Dichloromethane | 21.887 | 20.444 | 18.790 | 17.132 | 15.470 |

**Table S2.** Variations of polar free energy $\Delta G_a^p(T)$ $(kJ/mol)$ of different polar solvents adsorbed on SBR copolymer as a function of temperature.

| Temperature T(K) | 303.15 | 313.15 | 323.15 | 333.15 | 343.15 |
|---|---|---|---|---|---|
| Cyclohexane | 1.090 | 1.058 | 1.026 | 0.994 | 0.962 |

| | | | | | |
|---|---|---|---|---|---|
| Benzene | 1.723 | 1.696 | 1.669 | 1.642 | 1.615 |
| Toluene | 10.136 | 9.906 | 9.676 | 9.446 | 9.216 |
| Ethyl acetate | 13.590 | 12.963 | 12.336 | 11.709 | 11.082 |
| Ethanol | 12.346 | 9.772 | 7.198 | 4.624 | 2.050 |
| THF | 10.610 | 10.088 | 9.566 | 9.044 | 8.522 |
| Acetonitrile | 12.871 | 11.268 | 9.665 | 8.062 | 6.459 |
| Chloroform | 8.471 | 7.553 | 6.635 | 5.717 | 4.799 |
| Diethyl ether | 19.951 | 19.358 | 18.765 | 18.172 | 17.579 |
| Acetone | 13.690 | 12.899 | 12.108 | 11.317 | 10.526 |
| Dichloromethane | 7.405 | 6.455 | 5.505 | 4.555 | 3.605 |

**Table S3.** Variations of dispersive free energy $\Delta G_a^d(T)$ $(kJ/mol)$ of different solvents adsorbed on SBR copolymer as a function of temperature.

| Temperature T(K) | 303.15 | 313.15 | 323.15 | 333.15 | 343.15 |
|---|---|---|---|---|---|
| n-Pentane | 13.571 | 11.946 | 10.782 | 9.628 | 8.486 |
| n-Hexane | 16.059 | 14.136 | 12.759 | 11.393 | 10.041 |
| n-Heptane | 18.199 | 16.020 | 14.459 | 12.912 | 11.380 |
| n-Octane | 21.133 | 18.603 | 16.790 | 14.993 | 13.214 |
| n-Nonane | 22.974 | 20.224 | 18.253 | 16.300 | 14.366 |
| Cyclohexane | 14.661 | 12.906 | 11.648 | 10.402 | 9.168 |
| Benzene | 13.383 | 11.781 | 10.633 | 9.495 | 8.368 |
| Toluene | 14.922 | 13.135 | 11.855 | 10.587 | 9.331 |
| Ethyl acetate | 12.294 | 10.822 | 9.768 | 8.722 | 7.687 |
| Ethanol | 6.987 | 6.151 | 5.551 | 4.957 | 4.369 |
| THF | 10.703 | 9.422 | 8.504 | 7.594 | 6.693 |
| Acetonitrile | 6.493 | 5.716 | 5.159 | 4.607 | 4.060 |
| Chloroform | 12.588 | 11.081 | 10.001 | 8.931 | 7.871 |
| Diethyl ether | 12.411 | 10.925 | 9.861 | 8.805 | 7.761 |
| Acetone | 8.427 | 7.418 | 6.695 | 5.979 | 5.270 |
| Dichloromethane | 10.218 | 8.995 | 8.119 | 7.250 | 6.390 |
